# Postextubation Stridor: An Analysis of Incidence, Risk Factors, and Clinical Outcomes in Mechanically Ventilated Patients

**DOI:** 10.7759/cureus.116493

**Published:** 2026-09-18

**Authors:** Kenichi Nitta, Hiroshi Imamura, Hiroshi Kamijo, Katsunori Mochizuki, Yuichiro Kashima, Yasuaki Maeda, Kazutake Hasunuma, Takahito Katsuyama, Tomu Koyama, Chiaki Toida

**Affiliations:** 1 Advanced Emergency and Critical Care Center, Shinshu University Hospital, Matsumoto, JPN

**Keywords:** 30-day mortality, 60-day mortality, cuff-leak test, post-extubation stridor, reintubation, steroids

## Abstract

Background

Postextubation stridor (PES) is a potentially life-threatening complication following endotracheal intubation. However, detailed reports on its incidence and outcomes remain limited under standardized extubation protocols. In this study, we aimed to investigate the incidence, clinical characteristics, risk factors, and outcomes of PES, and evaluate the cuff leak test (CLT) for predicting PES.

Methods

This retrospective study included all patients who received mechanical ventilation for 48 hours or more at a single tertiary care center between January 2009 and December 2023. A comprehensive ventilator weaning and extubation protocol was implemented. Risk factors for PES were analyzed using univariate and multivariable logistic regression.

Results

Of 651 eligible patients, 22 (3.4%) developed PES. Among them, 14 (63.6%) were female, and 18 (81.8%) had passed the CLT. The reintubation rate was 36.4% (8/22). The 30-day and 60-day mortality rates were both 0% (0/22), and no deaths were attributable to PES. Treatment included epinephrine inhalation (13/22, 59.1%), noninvasive ventilation (11/22, 50.0%), and steroid administration (6/22, 27.3%). Multivariable analysis identified female sex (OR 3.65; 95% CI 1.49-9.47; p = 0.005) as the only factor independently associated with PES. Failed CLT did not reach statistical significance in the multivariable model (OR 3.21; 95% CI 0.85-9.84; p = 0.056). The CLT demonstrated high specificity (95.9%; 95% CI, 94.0-97.3%) but low sensitivity (18.2%; 95% CI, 5.2-40.3%) for predicting PES; however, these estimates are confounded by prophylactic corticosteroid administration in nearly all patients who failed the CLT.

Conclusions

PES remains a significant complication with high reintubation rates despite low overall incidence. Female sex was the only factor independently associated with PES in this cohort. The diagnostic performance of the CLT is confounded by the treatment paradox, and its low sensitivity necessitates vigilant post-extubation monitoring regardless of CLT results.

## Introduction

Endotracheal intubation is an essential procedure for respiratory support in critically ill patients. However, complications resulting from mechanical trauma to the larynx by the endotracheal tube (ETT) may lead to laryngeal edema (LE) [1-3]. As the luminal diameter narrows, local airflow accelerates and produces an audible inspiratory stridor after extubation - a phenomenon that serves as a bedside indicator of clinically significant LE [4]. Postextubation stridor (PES) is a potentially life-threatening event that often requires definitive airway control. To identify patients at risk of PES, airway patency assessment before extubation has been evaluated. According to the American Thoracic Society (ATS)/American College of Chest Physicians (CHEST) clinical practice guidelines, patients considered to be at elevated risk for PES include women, those intubated longer than six days, those with a relatively large ETT, those who underwent a traumatic intubation procedure, and those who required reintubation following an unplanned extubation [5]. Implementing structured extubation protocols that include systematic spontaneous breathing trials (SBTs) and explicit readiness criteria has been associated with shorter ventilation duration and fewer reintubation episodes [5-7]. However, detailed reports on the incidence and prognosis of PES under the use of such extubation protocols are scarce. Furthermore, each risk factor alone is insufficient to identify patients at high risk for PES [4], and investigations of physiological and descriptive data at extubation in PES patients remain limited. The purpose of this study was to investigate the incidence, clinical characteristics, risk factors, and outcomes of PES, and to evaluate the cuff leak test (CLT) for predicting PES under a standardized extubation protocol.

## Materials and methods

Study setting

This retrospective study included all patients who received mechanical ventilation (MV) under tracheal intubation in the Advanced Emergency and Critical Care Center of Shinshu University Hospital.

Patient selection

All patients who required MV under tracheal intubation for 48 hours or more between January 2009 and December 2023 at the study center were eligible. During this period, we implemented a comprehensive protocol for ventilator weaning and extubation for all consecutive adults [6]. Patients were excluded from the study if they were younger than 18 years of age, died while receiving MV, underwent tracheostomy, had self-extubation before or after fulfilling the conditions for SBT, were transferred to our center receiving MV, or had a do-not-resuscitate status. If patients received MV multiple times, only the first extubation attempt after MV for more than 48 hours was included. ETTs were selected with an inner diameter of 8 mm or less for males and 7 mm or less for females. The cuff pressure was intermittently measured and maintained at 25 cmH_2_O throughout MV in all patients.

The CLT was performed in two ways. In the qualitative method, the cuff was deflated while the ETT remained in situ and open to the atmosphere; an audible leak around the tube on auscultation indicated a positive (passed) result [8]. In the quantitative method, tidal volumes were measured with the cuff inflated and then deflated; a difference of 110 mL or more was classified as a pass, whereas a smaller difference was classified as a fail [9]. The CLT was performed either quantitatively or qualitatively at the discretion of the attending physician. If the CLT was passed, extubation was performed. In cases where the CLT failed, corticosteroids were administered prior to extubation. The choice of agent, dose, and timing was at the discretion of the attending physician and was not standardized during the study period. The CLT was repeated prior to extubation, and extubation was performed only if the CLT was passed. In the present analysis, the initial CLT result (i.e., the first CLT performed before any prophylactic intervention) was used for diagnostic performance evaluation and regression analysis.

A patient was identified as having PES by the presence of respiratory distress with high-pitched inspiratory wheezing. PES was assessed and recorded by attending intensivists and nurses, and decisions regarding medical treatment for PES were made by the attending intensivists.

Data collection

In all patients, the following parameters were recorded: age, sex, MV duration, CLT results, steroid administration for prevention of LE (dosage, timing, and rationale), and data characterizing all PES cases (including treatments). We investigated risk factors for failed CLT and PES, including female sex, intubation duration of more than six days, obesity (body mass index ≥ 30), acute brain injury, inhalation injury or burns affecting > 30% of total body surface area, upper airway infection or mass or trauma or surgery, moderate-to-severe facial or neck trauma or extensive facial surgery, and cervical spine fracture (C1-C5). The risk factors evaluated in this study were selected based on the risk stratification framework described by Kallet et al. [10]. The tool itself was not directly applied as a scoring system in the present study; rather, the individual risk factors listed by Kallet et al. were used as variables for analysis.

Outcome variables for PES patients included reintubation rates, hospital stay, and 30-day, 60-day, and in-hospital mortality. Additionally, the reason for steroid administration in patients with a passed CLT was investigated.

Statistical analysis

For continuous variables, the median and the 25th and 75th percentiles were calculated. Comparisons between the two groups were performed using the Mann-Whitney U test. Fisher's exact test was used for categorical variables. Univariate and multivariable logistic regression analyses were performed to identify factors associated with PES. The threshold for entry of variables into the multivariable model was p < 0.2. Odds ratios (ORs) and 95% confidence intervals (CIs) were also calculated. A p-value of < 0.05 was considered statistically significant. Statistical computations were carried out with EZR version 1.6-3 (Jichi Medical University, Saitama, Japan), an R-based platform designed for clinical and epidemiological research [11].

## Results

Twenty-two of 651 eligible patients developed PES, yielding an incidence of 3.4%. Fourteen of 22 PES patients (63.6%) were women. Among patients who developed PES, 18 (81.8%) had passed the CLT (Table 1).

**Table 1:** Clinical characteristics and outcomes of 22 patients with PES Data are presented as median (interquartile range) or number (percentage). CLT: cuff leak test; PES: postextubation stridor; BMI: body mass index; TBSA: total body surface area

Characteristic	PES (n=22)	Total (n=651)
Age (years)	64.5 (46.5-80.5)	70 (57-78)
Female	14 (63.6)	198 (30.4)
Passed CLT	18 (81.8)	621 (95.4)
Corticosteroid before extubation	6 (27.3)	48 (7.4)
PES Risk Factors		
Intubation >6 days	13 (59.1)	297 (45.6)
BMI ≥30	2 (9.1)	25 (3.8)
Acute brain injury	4 (18.2)	78 (12.0)
Inhalation injury or burns >30% TBSA	1 (4.5)	16 (2.5)
Cervical spine fracture (C1-C5)	2 (9.1)	30 (4.6)
Moderate-to-severe facial/neck trauma or extensive facial surgery	0 (0)	15 (2.3)
Upper airway infection/mass/trauma/surgery	0 (0)	18 (2.8)
Outcomes		
Reintubation	8 (36.4)	27 (4.1)
Hospital days	37.5 (30-49.3)	31 (20-49)
Hospital mortality	1 (4.5)	43 (6.6)
30-day mortality	0 (0)	12 (1.8)
60-day mortality	0 (0)	26 (4.0)

The reintubation rate was 36.4% (8/22), and the tracheostomy rate was 4.5% (1/22); the indication for tracheostomy was not PES-related (Table 2).

**Table 2:** Outcomes of 22 patients with PES Data are presented as median (interquartile range) or number (percentage). Continuous variables were compared using the Mann-Whitney U test. Categorical variables were compared using Fisher's exact test. Fisher's exact test does not produce a test statistic; only exact p-values are reported. Reintubation served as the grouping variable and was therefore not compared between groups. PES: postextubation stridor

Outcomes	Total (n=22)	Reintubation (n=8)	Non-reintubation (n=14)	Test statistic	p-value
Reintubation	8 (36.4)	-	-	-	-
Tracheostomy	1 (4.5)	1 (12.5)	0 (0)	-	0.364
Hospital days	37.5 (30-49.3)	37.5 (33-56.8)	38.5 (23.3-49.3)	U=66	0.516
Hospital mortality	1 (4.5)	0 (0)	1 (7.1)	-	1.000
30-day mortality	0 (0)	0 (0)	0 (0)	-	1.000
60-day mortality	0 (0)	0 (0)	0 (0)	-	1.000

The median hospital stay was 37.5 (30.0-49.3) days. Hospital mortality was 4.5% (1/22); the single hospital death was not attributable to PES but to the underlying disease. The 30-day and 60-day mortality rates were both 0% (Table 2). Sixteen of the 22 patients developed PES immediately after extubation; the remaining six developed PES at 30 minutes (n = 2), one hour (n = 2), and two hours (n = 2) post-extubation. Treatment for PES included epinephrine inhalation (13/22), steroid administration (6/22), noninvasive ventilation (NIV) (11/22), and nasal high-flow oxygen therapy (2/22) (Table 3).

**Table 3:** Treatments of 22 patients with PES Data are presented as number (percentage). Categorical variables were compared using Fisher's exact test. Fisher's exact test does not produce a test statistic; only exact p-values are reported. PES: postextubation stridor

Treatment	Total (n=22)	Reintubation (n=8)	Non-reintubation (n=14)	Test statistic	p-value
Epinephrine inhalation	13 (59.1)	4 (50.0)	9 (64.3)	-	0.662
Steroid administration	6 (27.3)	1 (12.5)	5 (35.7)	-	0.351
Noninvasive ventilation	12 (54.5)	3 (37.5)	9 (64.3)	-	0.378
Nasal high-flow oxygen therapy	2 (9.1)	1 (12.5)	1 (7.1)	-	1.000

No significant differences in treatment or hospital stay were observed between PES cases with and without reintubation (Tables 2-3). No significant difference in hospital stay was observed between the reintubation and non-reintubation groups (37.5 vs 38.5 days; U = 66, p = 0.516).

Predictive values of CLT and prophylactic effect of steroid administration for PES

As shown in Figure 1, 30 patients failed the CLT, comprising 4.6% of all patients.

**Figure 1:**
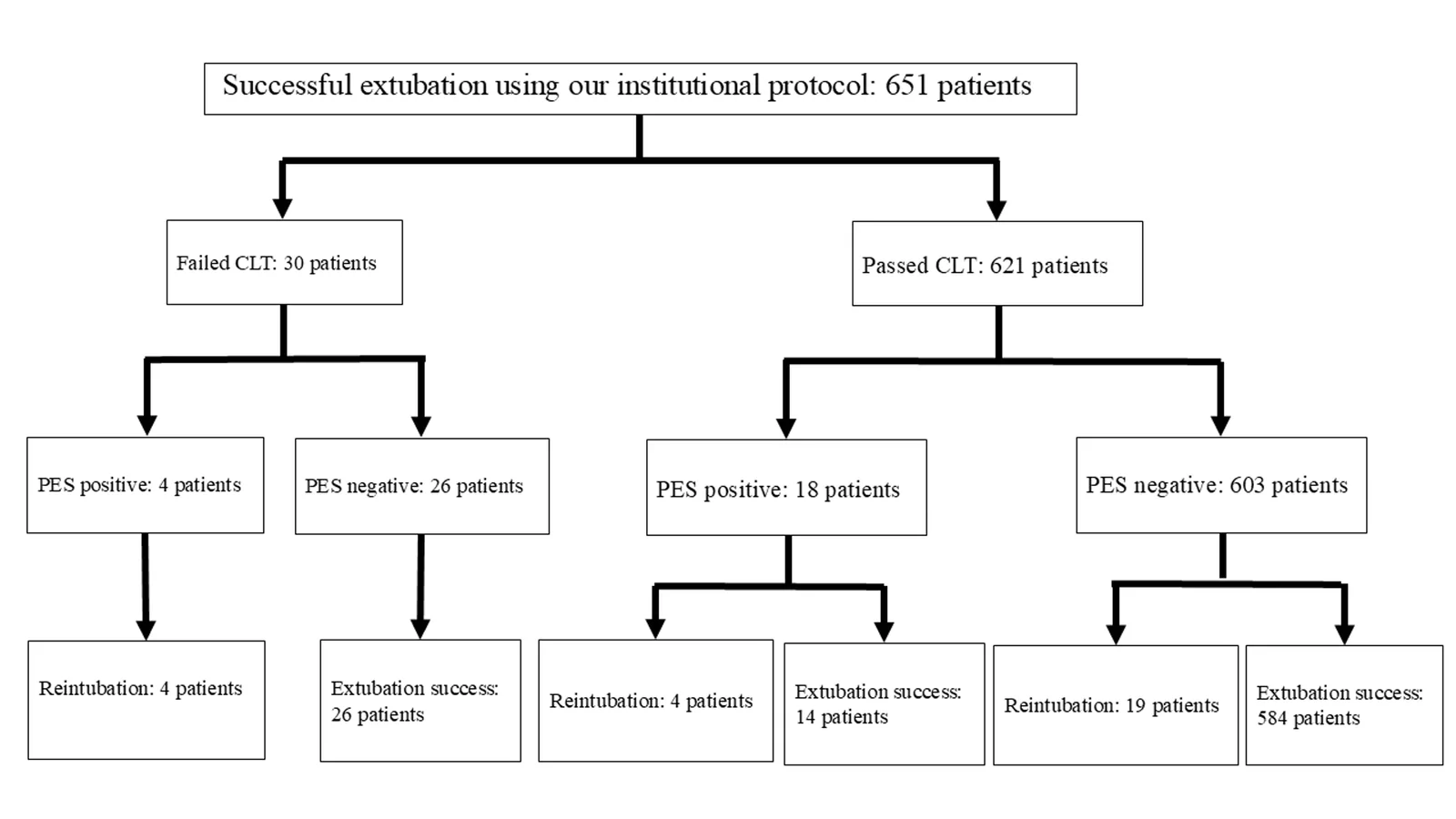
Flow chart of the study patients CLT: cuff leak test; PES: postextubation stridor

With the exception of one case, patients who failed the CLT received steroids prior to extubation for prevention of LE. Four of the 30 patients who failed the CLT developed PES, and all required reintubation, including the one patient who did not receive steroid treatment. Of the 621 patients who passed the CLT, 18 (2.9%) developed PES, and four of these 18 patients (22.2%) required reintubation. The sensitivity, specificity, positive predictive value (PPV), and negative predictive value (NPV) of the CLT for PES were 18.2% (95% CI 5.2-40.3%), 95.9% (95% CI 94.0-97.3%), 13.3% (95% CI 3.8-30.7%), and 97.1% (95% CI 95.5-98.3%), respectively.

A total of 48 patients received prophylactic corticosteroids prior to extubation: 29 who failed the CLT and 19 who passed the CLT but were considered at risk for PES based on clinical judgment. The corticosteroid regimens are summarized in Table 4.

**Table 4:** Types, doses, and timing of steroid prophylaxis for PES Regimens were selected at the discretion of the attending physician. Of the 48 patients, 29 had failed the initial CLT, and 19 had passed the CLT but received corticosteroids based on clinical judgment. mPSL: methylprednisolone; PES: postextubation stridor; CLT: cuff leak test

Steroid regimen	Number of patients
mPSL 20 mg every four hours, starting 12 hours before extubation	36
mPSL 20 mg four hours before extubation	3
mPSL 40 mg four hours before extubation	2
mPSL 125 mg every eight hours, starting 24 hours before extubation	2
mPSL 125 mg one hour before extubation	1
mPSL 40 mg immediately before extubation	2
Dexamethasone 3.3 mg four hours before extubation	1
Dexamethasone 3.3 mg immediately before extubation	1

Methylprednisolone was used in the majority of cases (46/48, 95.8%), with dexamethasone administered in the remaining two patients (4.2%). The most common regimen was methylprednisolone 20 mg every four hours starting 12 hours before extubation (36/48, 75.0%).

Nineteen patients who passed the CLT received prophylactic steroids for PES prevention. Among these 19 patients, indications for prophylactic steroids included prolonged intubation (n=9), cervical inflammation with suspected LE (n=6), airway burns (n=2), and other causes (n=2). The median duration of prolonged intubation was 15 (14-23) days.

Factors associated with PES

The results of univariate and multivariable analyses are presented in Table 5. In the multivariable model, female sex was the only factor that remained significantly associated with PES (OR 3.65; 95% CI 1.49-9.47; p = 0.005). Failed CLT showed a trend toward association but did not reach statistical significance (OR 3.21; 95% CI 0.85-9.84; p = 0.056) (Table 5).

**Table 5:** Univariate and multivariable logistic regression analyses for factors associated with PES The analysis included all 651 eligible patients (22 with PES and 629 without PES). The outcome variable was coded as 1 = PES and 0 = no PES. Variables with p < 0.2 in univariate analysis were entered into the multivariable model. Reference categories were as follows: male for sex, intubation ≤6 days, BMI <30, absence of each clinical condition, and passed CLT. OR and 95% CI were not calculated for "Moderate-severe facial/neck trauma or extensive facial surgery" and "Upper airway infection/mass/trauma/surgery" because there were no PES events in these groups (0/22), precluding estimation. OR: odds ratio; CI: confidence interval; BMI: body mass index; TBSA: total body surface area; CLT: cuff leak test; PES: postextubation stridor

Clinical characteristic	Univariate OR	95% CI	p-value	Multivariable OR	95% CI	p-value
Female	4.23	1.78-10.76	0.001	3.65	1.49-9.47	0.005
Intubation >6 days	1.75	0.75-4.31	0.202	-	-	-
BMI ≥30	2.64	0.41-9.79	0.209	-	-	-
Acute brain injury	1.67	0.47-4.61	0.367	-	-	-
Inhalation injury or burns >30% TBSA	1.96	0.11-10.35	0.523	-	-	-
Cervical spine fracture (C1-C5)	2.15	0.33-7.87	0.319	-	-	-
Moderate-severe facial/neck trauma or extensive facial surgery	-	-	-	-	-	-
Upper airway infection/mass/trauma/surgery	-	-	-	-	-	-
Failed CLT	5.15	1.41-15.03	0.005	3.21	0.85-9.84	0.056

## Discussion

Over the 14-year observation period, PES occurred in 22 of 651 eligible patients (3.4%). More than one-third of affected patients (8/22, 36.4%) required reintubation, and the majority (18/22, 81.8%) had demonstrated an adequate cuff leak before tube removal. Multivariable analysis identified female sex as the only factor independently associated with PES, while failed CLT did not reach statistical significance. No patient in the PES cohort died within 30 or 60 days.

PES is one cause of extubation failure and can potentially lead to reintubation. Although LE is clinically reflected by PES, stridor is not observed in all patients with LE [12]. The coexistence of respiratory distress and stridor is generally considered to indicate greater than 50% narrowing of the airway lumen [12]. The reported incidence of PES varies widely, with estimates ranging from 2.2 to 19.0% [2,3,10,13-20].

While PES refers to the audible clinical sign of inspiratory stridor, post-extubation laryngeal edema (PELE) encompasses the broader pathological finding of LE following extubation, which may or may not manifest as stridor [4,12]. Earlier studies have reported an incidence of PELE ranging from 11.0 to 36.8% [21-25]; however, the heterogeneity in reported rates likely stems from the absence of a universally accepted diagnostic standard, with each study applying its own criteria. Because stridor can be recognized at the bedside without specialized equipment, it has become the most commonly adopted endpoint for evaluating clinically relevant LE [12]. For this reason, the present study focused exclusively on PES.

The reported incidence of reintubation due to PES or PELE (or both) ranges from 1.0% to 12.5%, while reintubation is necessary in 11.8 to 100% of patients with PES or PELE [10, 13-18,20-25], which is consistent with our findings.

Earlier work by Epstein and Ciubotaru showed that patients reintubated for upper-airway obstruction had substantially better survival compared with those reintubated for other reasons [1]. In our study, the 30-day and 60-day mortality rates for PES patients were 0%. Although uncontrolled, this favorable outcome may be partly related to the use of a structured extubation protocol; however, a causal relationship cannot be established from this observational study.

Risk factors for PES

As shown in Figure 1, of the 621 patients who passed the CLT, 18 (2.9%) developed PES in this study. Therefore, we must recognize and address PES risk factors beyond failed CLT. The ATS/CHEST guidelines provide a weak recommendation that the CLT be reserved for high-risk patients, including those who have experienced traumatic intubation, those intubated for more than six days, those with a large ETT, women, or those reintubated after an unplanned extubation [5]. Among these factors, female sex is a well-established risk factor for both LE and PES [4,5,12-14]. Cadaveric measurements have demonstrated that women possess a narrower internal cricoid diameter and a shorter inter-cricoarytenoid distance than men, which may increase mucosal contact pressure when standard-sized tubes are used and thus predispose to ischemic injury [26], a finding corroborated by a recent computed tomography study [27]. In addition, bronchoscopic evaluation after prolonged intubation has revealed that tracheal granulation tissue and ulcerations can extend from the vocal cords to the ETT cuff site [24,28], suggesting that mucosal vulnerability is not limited to the subglottic region. In the present study, multivariable analysis identified female sex as significantly associated with PES (Table 5), consistent with these prior reports. Although failed CLT showed a trend toward association (p = 0.056), it did not reach statistical significance in the multivariable model. The small number of events and the resulting wide CIs limit the precision of these estimates.

The prediction of PES

Because the ETT itself prevents inspection of the glottic and subglottic structures, an indirect functional assessment - the CLT - was proposed as a means of detecting LE before tube removal [22]. A subsequent meta-analysis by Kuriyama et al. found that while the CLT’s specificity for airway obstruction after extubation approached 90%, its sensitivity remained in the range of 35-44% [29]. These characteristics make the test more reliable for ruling in clinically relevant edema - and thereby guiding prophylactic corticosteroid therapy - than for ruling it out [29]. In our study, the sensitivity was low, and the specificity was high; however, these estimates are substantially confounded by a treatment paradox. Nearly all patients who failed the CLT received prophylactic corticosteroids before extubation, which may have prevented PES in some cases. This intervention-after-testing pattern likely depressed the observed sensitivity and PPV while inflating the specificity and NPV relative to the CLT’s true untreated predictive performance. Therefore, close post-extubation monitoring should be maintained for all patients regardless of CLT results. It should be noted that the failure of the CLT to reach statistical significance in the multivariable model does not negate its clinical utility; the treatment paradox may have attenuated the observed association, and the small number of events limits the statistical power to detect a true effect.

Several methods exist to evaluate PES as alternatives to the CLT. Ultrasound evaluation of the larynx offers a noninvasive approach to estimating glottic patency. By comparing the air-column width before and after cuff deflation, this technique can quantify the degree of edema-related narrowing and has been explored as a complement to the CLT [16,19]. Nonetheless, a comprehensive review concluded that the PPV and overall diagnostic accuracy of ultrasonography for PES or PELE remain insufficient for routine clinical application [4].

Flexible laryngoscopy allows direct post-extubation assessment of laryngeal injury and edema severity through endoscopic visualization. Its pre-extubation utility, however, is restricted by the ETT, which blocks the view of the relevant structures [30]. An alternative approach monitors pressure in the aspiration channel proximal to the inflated cuff; a rising pressure signal indicates progressive subglottic narrowing without requiring cuff deflation. This method has been proposed as a technically simpler surrogate for the conventional CLT [31].

These methods complement or provide alternatives to the CLT, which has limitations in sensitivity and specificity for predicting severe airway obstruction or reintubation needs [16,19,30,31]. No single bedside assessment should be regarded as definitive in forecasting the outcome of extubation; clinical vigilance during the hours following tube removal remains indispensable.

Treatment after PES

The current treatment of choice for PES consists of intravenous steroids and nebulized epinephrine. Corticosteroids are believed to mitigate LE by attenuating the local inflammatory cascade and limiting microvascular leak. Evidence specifically addressing their therapeutic efficacy once stridor has developed, however, remains sparse, and an optimal dosing regimen has not been established. Extrapolating from preventive regimens in the literature, commonly cited doses include methylprednisolone 20 mg or dexamethasone 5 mg, continued for 24 to 48 hours following extubation [13,20,23].

Aerosolized epinephrine is administered on the premise that local α-adrenergic vasoconstriction will reduce mucosal swelling, although rigorous evidence confirming this benefit in adult PES is lacking. A dose of 1 mg diluted in 5 mL of normal saline has been suggested, but no consensus on optimal dosing exists [32]. Heliox, a blend of helium and oxygen, is an additional option that lowers airway resistance and alleviates the work of breathing. To date, no studies have evaluated its effectiveness specifically in adults with PES. In a pediatric cohort, heliox reduced the respiratory distress score by 38%, yet no significant impact on clinical outcomes was observed [33].

In contrast to its established role in averting first-time intubation, NIV applied after extubation failure has not demonstrated comparable benefit [34]. A landmark randomized trial found that continued NIV in this setting was associated with higher mortality, an observation attributed to postponement of definitive airway management [35]. Accordingly, routine reliance on NIV for post-extubation respiratory failure is not supported by current evidence, and clinicians should proceed to reintubation promptly when stridor-related respiratory compromise does not resolve rapidly.

However, since PES can range from mild to severe cases requiring immediate intubation, NIV may be worthwhile in mild to moderate cases. Indeed, the reintubation rate among our PES patients was 36.4%. Given the possibility of deterioration requiring reintubation, we remained prepared to perform reintubation without delay if PES worsened.

Limitations

This study has several limitations. First, because all data were derived from a single center using a retrospective approach, the results may be affected by selection bias and may not be readily applicable to other settings. Multicenter prospective validation is needed to confirm these observations. Second, PES diagnosis was based on clinical assessment by attending intensivists and nurses without standardized objective criteria. The absence of a uniform diagnostic protocol or scoring system for PES severity may have led to inter-observer variability and potential misclassification of cases. Third, the CLT methodology varied between quantitative and qualitative approaches at the discretion of attending physicians. This lack of standardization in CLT performance and interpretation may have influenced test results and subsequent clinical decisions, potentially introducing measurement bias. Furthermore, due to incomplete documentation over the 14-year study period, the proportion of patients assessed by each method could not be determined. Fourth, prophylactic steroid administration was not strictly protocolized beyond CLT results. The variation in steroid timing, dosing, and patient selection based on physician judgment may have affected the assessment of steroid use patterns. Additionally, the rationale for steroid administration in patients who passed the CLT was heterogeneous and physician-dependent. Because steroid administration was discretionary and non-randomized, the available data describe corticosteroid use rather than establish prophylactic efficacy. Fifth, the small number of PES cases (n = 22) limited statistical power for subgroup analyses. With only 22 events, the multivariable model had limited power to detect associations; notably, failed CLT did not reach statistical significance (p = 0.056) despite a clinically meaningful point estimate (OR 3.21). These results should be interpreted with caution, and larger studies are needed to clarify the independent contribution of failed CLT to PES risk. Sixth, the 14-year study period (2009-2023) may have been subject to temporal changes in clinical practice, staffing, and equipment that were not captured or adjusted for in the analysis. Finally, exclusion of patients who self-extubated and limiting ETT sizes to ≤8.0 mm for males and ≤7.0 mm for females may have eliminated high-risk patients, potentially underestimating the true incidence and severity of PES.

Despite these limitations, this study provides valuable real-world data on PES incidence, risk factors, and outcomes in a large cohort managed with a comprehensive extubation protocol. Future prospective multicenter studies with standardized assessment protocols are needed to validate our findings.

## Conclusions

This 14-year study of 651 patients revealed a 3.4% PES incidence, with 36.4% requiring reintubation. Female sex was the only factor independently associated with PES in this cohort. While the CLT helps identify candidates for prophylactic steroids, its diagnostic performance is confounded by the treatment paradox, and its limitations necessitate vigilant monitoring of all extubated patients. No PES-related 30-day or 60-day mortality was observed; however, whether the extubation protocol contributed to this favorable outcome remains unclear given the absence of a comparator group. Future research should explore more sensitive predictive tools and reassess the role of the CLT in high-risk patients.
